# A Closer Look at the Laboratory Impact of Utilizing ePlex Blood Culture Identification Panels: a Workflow Analysis Using Rapid Molecular Detection for Positive Blood Cultures

**DOI:** 10.1128/spectrum.01796-22

**Published:** 2022-09-07

**Authors:** Giannoula S. Tansarli, Kimberle C. Chapin

**Affiliations:** a Department of Pathology, Rhode Island Hospital, Providence, Rhode Island, USA; b The Warren Alpert Medical School of Brown University, Providence, Rhode Island, USA; Johns Hopkins Hospital

**Keywords:** sepsis, bacteremia, bloodstream infection, molecular diagnostics, rapid identification, antimicrobial resistance, molecular identification

## Abstract

Rapid identification of pathogens is critical in bloodstream infections. We evaluated the diagnostic performance of the GenMark Dx ePlex blood culture identification (BCID) panels and the adoption of the ePlex system into the clinical laboratory workflow. Nonduplicate remnant specimens of positive blood cultures were prospectively tested using ePlex panels between January and March 2020. A total of 313 unique positive blood culture specimens were tested. The identified organisms consisted of 98 Gram-negative rods (GNR), 90 Gram-positive cocci (GPC) in clusters, 62 GPC in chains, 21 Gram-positive rods, and 20 yeasts; 22 organisms were off panel. The positive percent agreement was 100% across all organisms tested after discordancy resolution, while the negative percent agreement was 100% across all targets except Corynebacterium spp., where it was 99.7%. The ePlex BCID panels accurately detected 5 pan targets and 42 antimicrobial resistance gene markers, including 31 *mecA*, 4 *vanA*, 6 CTX-M, and 1 KPC gene. The median times to result were calculated as 2.5 h for Xpert MRSA/SA in GPC in clusters, 9.5 h for Accelerate Pheno (identification and susceptibility) in GNR, 6 h for peptide nucleic acid fluorescent *in situ* hybridization [PNA-FISH] in yeasts, 27 h for the latex agglutination test in S. aureus, 29 h for Lancefield serotyping in GPC in chains, and 29 h for Vitek-MS in GNR. In our laboratory, the ePlex panels could substantially reduce the time to result for bloodstream infection (BSI) caused by Streptococcus spp., Enterococcus spp., and Candida spp. The highly accurate ePlex panels can help streamline laboratory efficiency in the blood bench workflow, reducing the time to result for identification of BSI pathogens.

**IMPORTANCE** Sepsis is a leading cause of morbidity and mortality worldwide. Rapid identification of the causative agent is of critical importance for the prompt initiation of the appropriate antibiotic treatment. In this study, we evaluated the diagnostic performance of the GenMark Dx ePlex blood culture identification (BCID) panels and their adoption into the clinical laboratory workflow. We prospectively tested 313 blood culture isolates and found that ePlex BCID panels had a positive percent agreement of 100% across all organisms tested after discordancy resolution. The negative percent agreement was 100% across all targets except Corynebacterium spp., where it was 99.7%. This new rapid technology (turnaround time of ~90 min) can help streamline laboratory efficiency in the blood bench workflow, reducing the time to result for identification of BSI pathogens. Adoption should be individualized based on the needs of the patient population and capabilities of the laboratory.

## INTRODUCTION

Sepsis is a leading cause of morbidity and mortality worldwide. In the United States, it is the most common cause of in-hospital mortality and health care costs attributed to sepsis every year are over $24 billion (1). Mortality rates can range anywhere between 25 and 70%, with the highest being among patients with infections due to multidrug-resistant organisms (2). Sepsis is a medical emergency, and it has been found that for every hour of delay in appropriate treatment, the survival rate decreases by 7.6% (3). Rapid identification of the causative agent is of critical importance for the prompt initiation of the appropriate antibiotic treatment. Over the last decade, many different diagnostic methods, molecular and nonmolecular (e.g., matrix-assisted laser desorption ionization–time of flight mass spectrometry [MALDI-TOF MS] [4], peptide nucleic acid fluorescent *in situ* hybridization [PNA-FISH] [5], T2 magnetic resonance [6], and Accelerate PhenoTest [7]), were developed and significantly shortened the turnaround time for positive blood cultures. Likewise, syndromic-panel-based testing has been a recent revolution in the diagnosis of infectious diseases (8–10). In bloodstream infections (BSIs), these molecular panels can simultaneously detect and identify the most common Gram-positive (GP)/Gram-negative (GN) bacteria and fungi directly from positive blood cultures (11–13). These FDA-approved multiplex panels provide identification of the organism and detect some of the most common resistance genes (e.g., *mecA*, *vanA*, *vanB*, CTX-M, KPC, MBL, and OXA-48 genes) in 1 to 2.5 h.

**FIG 1:**
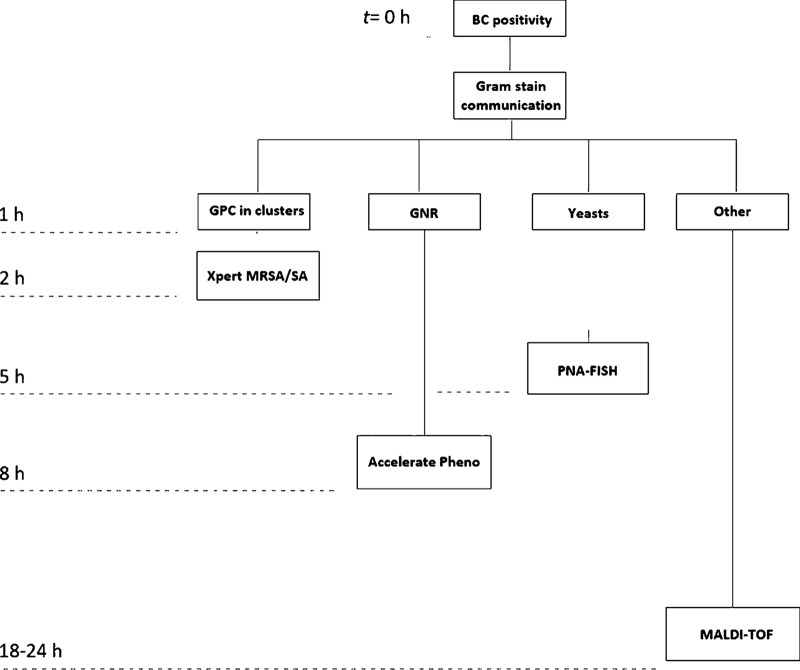
mRDT algorithm for positive blood culture (BC) in our laboratory.

The institutional laboratories at Rhode Island Hospital and The Miriam Hospital process approximately 20,000 blood cultures annually, with positivity rates of 9 to 16%, using a multitude of different laboratory processes to identify bacteria and fungi. In this study, we sought to evaluate the performance of the GenMark Dx ePlex blood culture identification (BCID) panels (Carlsbad, CA) for Gram-positive bacteria, Gram-negative bacteria, and yeasts and examine how this new molecular rapid diagnostic test (mRDT) could streamline diagnostic testing in the microbiology laboratory. The mRDT algorithm implemented in our laboratory for positive blood cultures is depicted in Fig. 1.

## RESULTS

In total, 313 unique prospective positive blood culture specimens were Gram stained and tested with the appropriate ePlex BCID panels between January and March 2020. Eighteen of 313 (5.8%) cultures were collected from pediatric patients, while the rest were collected from adult patients. The organisms identified by the ePlex (*n* = 291) consisted of the following: 98 (33.7%) Gram-negative rods (GNR), 90 (30.9%) Gram-positive cocci (GPC) in clusters, 62 (21.3%) GPC in chains, 21 (7.2%) Gram-positive rods, and 20 (6.9%) yeasts. While samples were tested prospectively, to achieve an organism variety to test all panels, not all positive consecutive cultures were tested. The normal distribution in the laboratory annually is 45% GPC in clusters, 25% GNR, 20% GPC in chains, 8% Gram-positive rods, and 2% yeasts.

### Performance.

The positive percent agreement was 100% across all organisms tested after discordancy resolution, while the negative percent agreement was 100% across all targets except Corynebacterium spp., where it was 99.7% (Table 1). In addition, there were 22 organisms (7%) that were off panel and were not detected by the ePlex BCID panels (Table 2). Also, the ePlex detected 5 pan targets: 3 pan Gram-positive, 1 pan Gram-negative, and 1 pan *Candida*. Additionally to the individual targets, the BCID-GP and BCID-GN Panels contain two pan targets each (BCID-GP: pan Gram-negative, pan Candida; BCID-GN: pan Gram-positive, pan Candida). Moreover, the ePlex BCID-GP and BCID-GN panels detected 42 antimicrobial resistance gene markers: 31 *mecA*, 4 *vanA*, 6 *bla*_CTX-M_, and 1 *bla*_KPC_. The detailed results on the resistance markers detected are presented in Table 3. Regarding mixed infections, 15 were detected by the ePlex panels, and 14 of them were confirmed by standard-of-care (SOC) testing; mixed infections were due to two Gram-positive organisms, one Gram-positive/one Gram-negative, two Gram-negative, or two fungal organisms. The one discrepant result was a culture with Micrococcus and Corynebacterium spp. detected by the ePlex but only Micrococcus identified by SOC testing. Corynebacterium was not detected during discordancy analysis either, suggesting that the ePlex result was a false positive.

**TABLE 1:** Performance characteristics of the ePlex BCID panels

Panel, organism(s) detected	No. with indicated result/no. of specimens tested (% agreement)	
	Positive	Negative
Gram-negative rods		
Escherichia coli	44/44 (100)	269/269 (100)
Klebsiella pneumoniae group	24/24 (100)	289/289 (100)
Pseudomonas aeruginosa	7/7 (100)	306/306 (100)
Proteus mirabilis	3/3 (100)	310/310 (100)
Serratia marcescens	3/3 (100)	310/310 (100)
Klebsiella oxytoca	3/3 (100)	310/310 (100)
Enterobacter cloacae complex	2/2 (100)	311/311 (100)
Klebsiella (formerly Enterobacter) aerogenes	2/2 (100)	311/311 (100)
Stenotrophomonas maltophilia	1/1 (100)	312/312 (100)
Acinetobacter baumannii	1/1 (100)	312/312 (100)
Citrobacter koseri	1/1 (100)	312/312 (100)
Haemophilus influenzae	1/1 (100)	312/312 (100)
Bacteroides fragilis	2/2 (100)	311/311 (100)
Fusobacterium nucleatum	3/3 (100)	310/310 (100)
Fusobacterium necrophorum	1/1 (100)	312/312 (100)
Gram-positive cocci in:		
Clusters		
Staphylococcus aureus		
Methicillin susceptible	35/35 (100)	278/278 (100)
Methicillin resistant	7/7 (100)	306/306 (100)
Coagulase-negative staphylococci	37/37 (100)	276/276 (100)
Staphylococcus lugdunensis	1/1 (100)	312/312 (100)
Micrococcus spp.	10/10 (100)	303/303 (100)

Chains		
Streptococcus pneumoniae	10/10 (100)	303/303 (100)
Streptococcus anginosus group	8/8 (100)	305/305 (100)
Streptococcus pyogenes (group A)	3/3 (100)	310/310 (100)
Streptococcus agalactiae (group B)	5/5 (100)	308/308 (100)
Other streptococci (Streptococcus spp.)	18/18 (100)	295/295 (100)
Enterococcus faecalis	15/15 (100)	298/298 (100)
Enterococcus faecium	3/3 (100)	310/310 (100)
Gram-positive rods		
Corynebacterium spp.	12/12 (100)	301/302 (99.7)
Cutibacterium acnes	4/4 (100)	309/309 (100)
Bacillus subtilis group^*a*^	2/2 (100)	311/311 (100)
Bacillus cereus group	1/1 (100)	312/312 (100)
Lactobacillus spp.	2/2 (100)^*b*^	311/311 (100)

Yeasts		
Candida albicans	9/9 (100)	304/304 (100)
Candida parapsilosis	4/4 (100)	309/309 (100)
Candida glabrata	2/2 (100)	311/311 (100)
Candida tropicalis	2/2 (100)	311/311 (100)
Candida krusei	1/1 (100)	312/312 (100)
Candida lusitaniae	2/2 (100)	311/311 (100)
Pan targets		
Pan Gram-positive	3	
Pan Gram-negative	1	
Pan Candida^*c*^	1	

a There were 2 Bacillus subtilis group organisms detected by the ePlex; one of them was verified by MALDI-TOF MS, but the other was called as Bacillus spp. not *anthracis* by the standard of care based on Gram stain, colony morphology, and biochemicals because MALDI-TOF MS failed twice. Also, there were 2 specimens positive for Bacillus spp. not *anthracis* whose results by the ePlex BCID were “not detected” that might have been species other than B. subtilis or B. cereus, most likely blood culture contaminants from normal skin flora.

b There was 1 Lactobacillus spp. that was not detected by the BCID-GP, but it was not counted as a false negative because the technologist selected to run a BCID-GN because of misreading the Gram stain as Gram-negative rods instead of Gram-positive rods.

c The pan *Candida* detected was done when using the BCID-GP.

**TABLE 2:** Organisms growing from cultures but not included as targets on the ePlex BCID panels

Panel, off-panel organism(s) found	No. of specimens
Gram negative	
Aeromonas hydrophila	1
Bacteroides thetaiotaomicron	1
Campylobacter jejuni	1
Capnocytophaga canimorsus	1
Enhydrobacter aerosaccus	1
Moraxella spp.	2
Pasteurella multocida	1
Raoultella ornithinolytica	1
Sphingomonas paucimobilis	1
Veillonella spp.	1
Gram positive	
Actinomyces spp.	2
Aerococcus viridans	1
Anaerococcus prevotii	1
Bacillus spp. *not anthracis*	1
Clostridium spp.	1
Dermacoccus nishinomiyaensis	1
Finegoldia magna	2
Granulicatella adiacens	1
Rothia mucilaginosa	1

**TABLE 3:** Antibiotic resistance mechanisms

Antibiotic resistance mechanism, organism(s)	No. of specimens with result that were:	
	Detected by ePlex BCID	Confirmed by SOC testing
*mecA*	31	31
Staphylococcus aureus	24	24
Coagulase-negative staphylococci	25	24^*a*^
*vanA*	4	4
Enterococcus faecium	3	3
Enterococcus faecalis	1	1
*bla*_CTX-M_	6	6
Escherichia coli	5	5^*b*^
Klebsiella pneumoniae	1	1
*bla*_KPC_	1	0^*c*^
Enterobacter cloacae complex	1	0^*c*^

a There was one specimen positive for Staphylococcus epidermidis, *mecA*, by ePlex which was identified as methicillin-susceptible Staphylococcus aureus by latex agglutination test. Upon discrepancy analysis, it was verified as Staphylococcus epidermidis.

b Two E. coli isolates that were extended-spectrum β-lactamase positive [ESBL(+)] by Vitek 2 and confirmatory disk test were not detected as such by the ePlex BCID-GN. The isolates were resistant to third-generation cephalosporins, and thus, it is possible they produced an ESBL other than *bla*_CTX-M_.

c There was 1 Enterobacter cloacae complex that was identified as *bla*_KPC_ by the ePlex BCID-GN but as susceptible to carbapenems when it was tested with Sensititre. Sensititre gave the same results upon repeat for confirmation. It is possible that the carbapenemase-encoding gene (KPC) was not expressed (25).

### Discordancy analysis.

Results were discordant between the ePlex BCID panels and SOC testing in nine cultures, which were further tested by additional methods. The results of the discordancy analysis are presented in Table 4.

**TABLE 4:** Discordancy resolution

SOC testing result	No. of specimens	ePlex result(s)	Resolution of discrepancy
CoNS, oxacillin susceptible	2	*mecA* detected	*mecA* confirmed by secondary and TOPO cloning, produced colonies with *mecA* morphology
E. faecalis	1	E. faecalis, S. epidermidis, *mecA*	S. epidermidis recovered upon reculture and *mecA* confirmed by secondary PCR
Micrococcus spp.	1	Pan Gram negative (BCID-GP), no targets detected (BCID-GN)	Sequencing identified Micrococcus antarcticus^*a*^ and Arthrobacter pascens^*b*^
Bacillus spp. not *anthracis*	1	No targets detected	Sequencing identified Bacillus niabensis^*a*^
Bacillus spp. not *anthracis*	1	Bacillus subtilis group	Sequencing identified Bacillus licheniformis
Lactobacillus spp.	1	No targets detected	Sequencing identified Lactobacillus crispatus^*a*^
Micrococcus spp.	1	Micrococcus spp., Corynebacterium spp.	False positive: Corynebacterium spp. not recovered upon resubculture from the bottle
Moraxella spp.	1	No targets detected (BCID-GP and BCID-GN)	False negative: sequencing identified Moraxella osloensis

a Detected organism not predicted based on inclusivity of target and *in silico* analysis.

b The pan Gram-negative assay is designed to detect a broad range of Gram-negative organisms.

All pan targets were verified by culture. The pan Gram-negative target was a case of A. baumannii that was read as GPC, and thus, BCID-GP was selected and tested. After the pan Gram-negative call was detected, BCID-GN was tested and A. baumannii was detected. The pan Candida target was positive in a patient with a polymicrobial (>3 bacterial pathogens) bloodstream infection whose Gram stain from the blood culture bottle and subculture did not show or grow yeasts. When the specimen was tested with BCID-FP, C. albicans was detected and, additionally, C. albicans grew on the chromogenic agar upon discordancy analysis.

### Potential impact of the ePlex BCID panels on the laboratory workflow.

**FIG 2:**
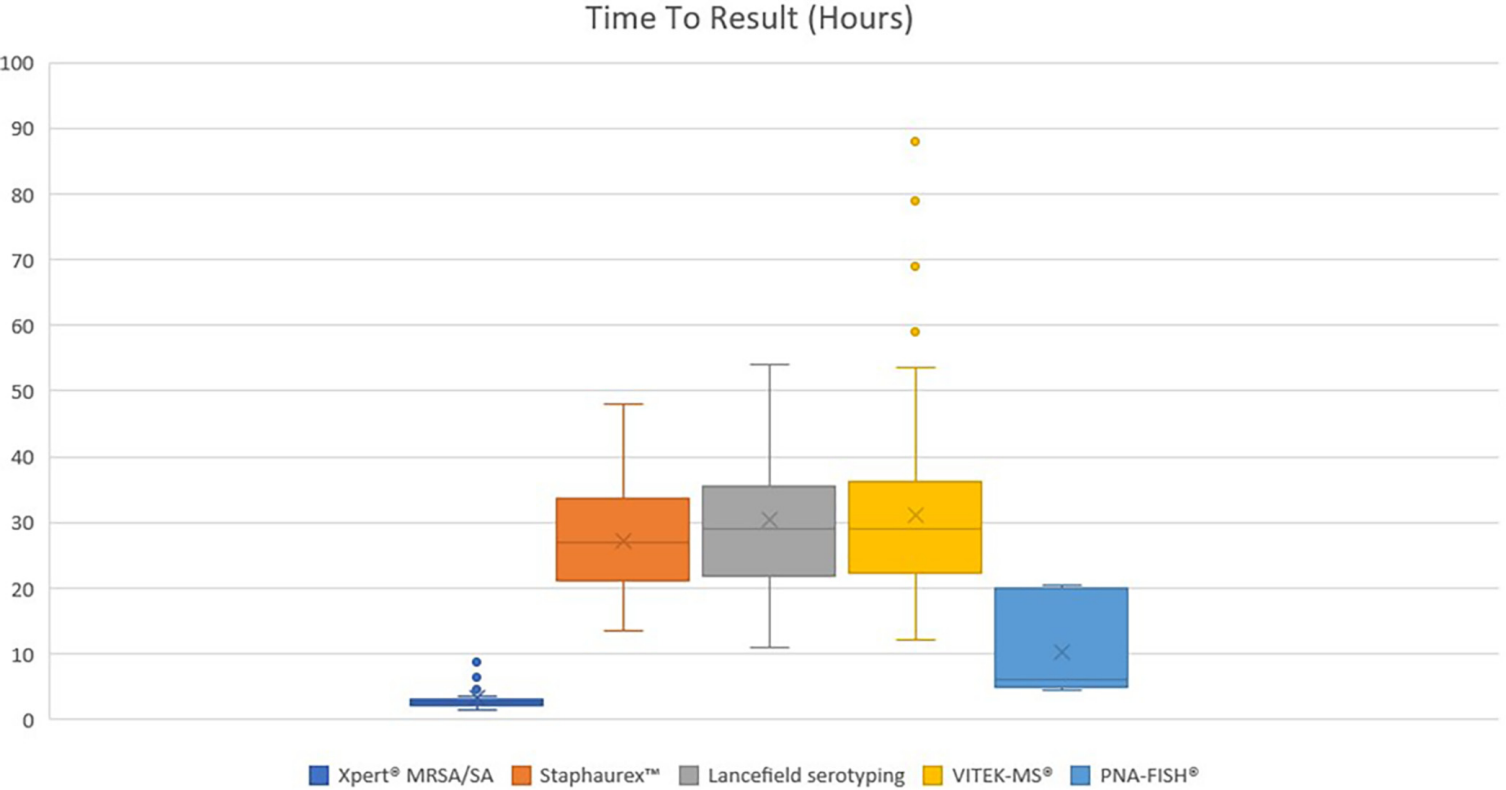
Time to result. Defined in this graph as the time from Gram stain reading to calling the final identification of the organism to the provider. Median time and interquartile range for each mRDT are shown.

The median times to result (identification [ID]) for each assay from our laboratory data were the following: 2.5 h (interquartile range [IQR], 0.8 h) for Xpert MRSA/SA, 6 h (IQR, 15 h) for PNA-FISH, 27 h (IQR, 12.5 h) for latex agglutination test for S. aureus, 29 h (IQR, 15 h) for Lancefield serotyping, and 29 h (IQR, 13.5 h) for MALDI-TOF MS (for GNR). These results are shown in a box and whisker chart in Fig. 2.

In our laboratory, the biggest reduction in time to result from the use of the ePlex BCID panels could be seen in BSI caused by GPC in chains (i.e., Enterococcus spp. and Streptococcus spp.), for which we use assays that require colony growth (e.g., MALDI-TOF MS and Lancefield serotyping), as well as in candidemia, where we use PNA-FISH for identification. A time comparison between the ePlex BCID panels and Accelerate Pheno may not be suitable because Accelerate Pheno provides both ID and antimicrobial susceptibility testing (AST), and although the ID only is usually ready at 2 h, according to our institutional policy, we release it along with the AST. The median time to result (ID plus AST) for Accelerate Pheno was 9.5 h (IQR, 2.5 h). Likewise, comparison between ePlex BCID-GP and Xpert MRSA/SA is best performed with real-life (not hypothetical) data for both assays because the turnaround times are so similar.

### Advantages and disadvantages of the ePlex BCID panels.

**FIG 3:**
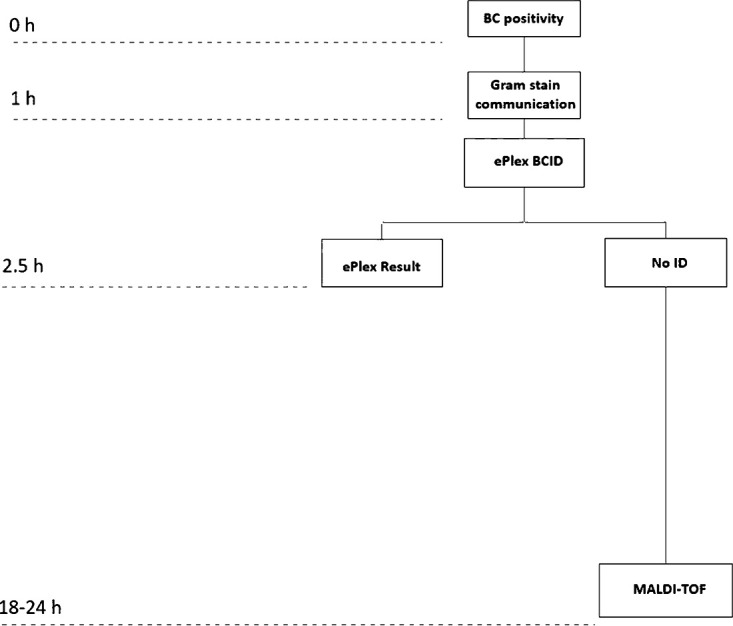
Hypothetical BC algorithm using ePlex panels as the sole rapid ID method.

A hypothetical algorithm using ePlex BCID panels as the only rapid ID method for positive blood cultures is depicted in Fig. 3. Adoption of the ePlex BCID panels into the laboratory workflow should be individualized based on the needs of the patient population and the capabilities of the laboratory after taking into consideration specific parameters. In Table 5, the most important advantages and disadvantages of the ePlex are presented in comparison to those of other mRDTs used in the laboratories at Rhode Island Hospital and the Miriam Hospital.

**TABLE 5:** Advantages and disadvantages of ePlex BCID panels compared to other mRDTs

Parameter	Item
Advantages	Faster results than for Accelerate Pheno and PNA-FISH
	Broader panel targets for GP/GN bacteria and yeasts/fungi
	Equal or less technologist time
	Specific panels for all targets (GP/GN bacteria and yeasts/fungi), giving the potential to streamline training to one mRDT method, to have one IQCP, and to free technologist time and reduce external control needs
	Antimicrobial resistance gene markers for GP/GN bacteria
	FDA cleared for multiple blood culture bottle types
	Pan targets to assist in polymicrobial cultures or difficult-to-read Gram stains
Disadvantages	Higher cost per test than for Xpert MRSA/SA and PNA-FISH
	No AST (compared to Accelerate Pheno)

## DISCUSSION

The ePlex BCID panels provided highly accurate identification results for GP and GN bacteria, as well as for yeasts, and these findings are consistent with previous studies (11, 14, 15). Importantly, this system may reduce substantially the time to final identification for most pathogens encountered in our population compared to the time using a combination of other testing methods.

During the 3-month period, we had the opportunity to test a variety of GP and GN bacteria, as well as various species of Candida, and the ePlex BCID panels provided accurate identification for the vast majority of the blood cultures. The broad coverage of the panels helped to rapidly and accurately identify organisms that previously required days of incubation and selective agar media. Pathogens with resistance to many antibiotics, such as Stenotrophomonas maltophilia, that usually take at least 24 to 48 h to be identified with most available methods were identified within ~90 min. Similarly, BCID-GN accurately identified all anaerobic Gram-negative rods, such as Fusobacterium spp. or Bacteroides fragilis, which take days for full identification with all other available methods. In comparison to similar molecular blood culture panels, such as Verigene BCID (Nanosphere, Northbrook, IL) for GP and GN bacteria (16) or BioFire FilmArray BCID2 for GP, GN, and yeasts (17), the broader menu offered on the ePlex enables the rapid identification of some less common but severe blood pathogens (i.e., Fusobacterium spp., Morganella morganii, Cronobacter sakazakii, and Candida lusitaniae), although it should be noted that BioFire BCID2 is one panel for Gram-positive and Gram-negative bacteria and yeasts instead of three different panels. Moreover, the ePlex pan target feature is not offered by any other blood culture identification platform, and this feature can prove particularly helpful in polymicrobial infections and, more importantly, in the not uncommon cases of Gram stain misreading. In our study, the only case where a Gram stain was misread was a case with A. baumannii reported initially as GPC, but the pan Gram-negative target on the BCID-GP was positive, indicating that a BCID-GN should be run instead, highlighting the utility of these targets being present when utilizing a Gram stain-driven approach for testing. A. baumannii bacteria are known to retain crystal violet and not destain sometimes, and also, they may become coccoid (instead of rods) at certain phases of the cell cycle, which explains the Gram stain misreadings.

Many laboratories, such as the one at Rhode Island Hospital, that only use a Gram-positive molecular test method for GPC in clusters may have missed an opportunity to rapidly identify GPC in chains, which accounted for 30% of our GPC. Since enterococci are treated differently than other streptococci, it is very important for the clinician to have a rapid identification and initiate the appropriate empirical treatment (18). The BCID-GP differentiated Enterococcus spp. (including species differentiation for Enterococcus faecium and Enterococcus faecalis) from Streptococcus pneumoniae, group A or group B Streptococcus, or nonsignificant alpha-hemolytic streptococci without a problem. It also performed exceptionally with mixed fungemia, accurately identifying more than one Candida spp. in a single bottle (i.e., Candida albicans and Candida parapsilosis). While no cases of Candida auris were identified during the study, the BCID-FP does identify this pathogen. The BCID-GP did help in significantly identifying the majority of blood culture contaminants, including most coagulase-negative staphylococci (CoNS), Corynebacterium spp., and Micrococcus spp. (19). It did miss a few Bacillus spp. of environmental origin contaminating the tops of the blood culture bottles because those were not part of the Bacillus cereus or Bacillus subtilis group calls, as well as one species of Lactobacillus that was not included in the genus call of Lactobacillus on the BCID-GP. For Bacillus spp. in particular, no target detection by the BCID-GP in a single positive bottle with a Gram stain morphology looking like Bacillus spp. would indicate a probable blood culture contaminant. Because Bacillus spp. can occasionally be misread as GNR due to overdecolorization of the rods, leading to inappropriate initiation of antibiotics, it would be particularly helpful if the pan Gram-positive target included the most common environmental Bacillus spp. too.

Another benefit of the ePlex BCID panels is the antimicrobial resistance markers. The BCID-GN and BCID-GP panels include 10 targets for some of the most common and emerging antimicrobial resistance genes, such as *mecA*, *mecC*, *vanA*, CTX-M, KPC, and MBL genes. The presence of these genes on the microbiology report can prove to be very helpful (15), particularly in geographic areas with a high prevalence of antimicrobial resistance, since the detection of and reporting of a gene encoding a carbapenemase in a Gram-negative rod from a blood culture will result in prompt initiation of the appropriate antimicrobial therapy (15, 20). On the flip side, knowing the resistance profile of every single organism may not always be helpful. For instance, reporting a *mecA* in a CoNS out of a single blood culture set may create confusion for the health care provider who may not be an infectious diseases specialist. For this reason, reporting of the resistance genes should be based on the epidemiological characteristics of each area and in collaboration with the antibiotic stewardship team. Notably, the ePlex system allows users to add customizable comments that can aid in clarifying results. Interestingly, it has been suggested that mRDT with an antibiotic stewardship program resulted in the highest savings per death averted compared to other approaches and was more cost effective than without stewardship (21).

The ePlex BCID panels can considerably reduce the time to result compared to those of other assays, as well as save technologist time compared to the time required for assays with more complex protocols, such as PNA-FISH. Compared to Accelerate Pheno, the cost per test and time to identification may be lower for ePlex, although Accelerate Pheno also provides phenotypic AST for some of the antibiotics most commonly used in bacteremia if the bacterium is included on the Pheno panel. Moreover, the use of BCID-GP can help to quickly deescalate antimicrobial therapy in specific patients (22), which may result in clinical and indirect economic benefits. However, the panel should be used wisely in laboratories with high blood culture contamination rates because of the relatively high direct cost as a molecular test compared to traditional culture.

In laboratories where more than one mRDT is available, implementation of the ePlex BCID panels for all positive blood cultures could simplify the laboratory workflow and operations while allowing the adoption of a single platform, consolidating multiple facets of the blood bench. Utilization of the ePlex BCID panels in place of a multitest algorithm in the clinical laboratory highlights many advantages, including having a single individualized quality control plan (IQCP) at the blood bench as opposed to taking the time to implement and keep up IQCPs for all the different tests that are currently employed. Along these lines, it is important to consider that each piece of instrumentation requires its own set of quality controls, proficiency testing, and competency for quality assurance, which may be overcome when one mRDT platform is implemented to cover a very broad range of pathogens.

### Conclusion.

This study showcases the excellent performance of the ePlex BCID panels for coverage and identification of BSI-causing organisms in a large academic institution and provides insight as to how and where a laboratory can potentially save time and costs by consolidating platforms to obtain IDs and antimicrobial resistance marker information for positive blood cultures. Laboratories where no mRDTs are currently in use for blood cultures may further benefit from this type of approach. Due to this being a single-site study, diagnostic stewardship can justify the implementation based on the needs of the patient population, antimicrobial stewardship initiatives, test volume of the laboratory, and staffing.

## MATERIALS AND METHODS

### Study design.

Nonduplicate remnant specimens of positive blood cultures (Thermo Scientific VersaTREK Redox medium) were prospectively tested, based on the Gram stain results, with the appropriate ePlex BCID panels in the Microbiology Laboratory at Rhode Island Hospital from January 2020 through March 2020. Only one isolate per patient was included. To ensure there would be a variety of organisms during the 3 months, all blood isolates were tested consecutively except for common blood culture contaminants (e.g., Bacillus spp., Corynebacterium spp., Cutibacterium acnes, and coagulase-negative staphylococci). We considered an organism as a possible contaminant primarily based on the Gram stain morphology and the number of positive blood culture sets. For example, when Gram-positive bacilli consistent with Bacillus spp., pleomorphic club-shaped Gram-positive rods consistent with diphtheroids, or “spidery” Gram-positive rods consistent with Cutibacterium acnes were seen on the Gram stain from a single positive set or bottle, it was hypothesized that this was most likely a contaminant. In some cases, a preliminary identification of the organism (e.g., coagulase-negative staphylococci) was available prior to running the ePlex, which helped to determine whether an organism was a blood culture contaminant.

The ePlex BCID panel results were not released to the provider at the time, as the instrument was being used only for research purposes. Currently, all three panels have 510(k) clearance from the FDA. The study was approved by the Institutional Review Board of Rhode Island Hospital, and because of the use of remnant specimens and nature of the study, no informed consent was required.

### Standard microbiological procedure for positive blood cultures.

Standard-of-care (SOC) testing for the blood bench includes Gram staining of the positive bottle and subsequent steps dependent on this result, including molecular rapid diagnostic testing (mRDT) and culture for identification (ID) and antimicrobial susceptibility testing (AST). mRDTs are routinely performed depending upon the Gram stain result. More specifically, for GPC in clusters, first patient positive, the Xpert MRSA/SA test (Cepheid, Sunnyvale CA) is performed to differentiate Staphylococcus aureus (methicillin resistant or methicillin susceptible) from common probable contaminants like CoNS and micrococci. When an mRDT cannot be used for GPC in clusters (the Xpert MRSA/SA test is not approved for use with anaerobic bottles), a latex agglutination test (Thermo Scientific Staphaurex) is performed for S. aureus from the colony. Additionally, a bacitracin disk is dropped onto the blood agar plate every time GPC in clusters are seen on Gram stain, for identification of Micrococcus spp. Lancefield serotyping (Thermo Scientific Remel PathoDX) for beta-hemolytic streptococci and the optochin disk test for alpha-hemolytic streptococci are performed along with MALDI-TOF MS for GPC in chains. No mRDT is offered at the laboratory for GPC in chains. For Gram-negative rods, the Accelerate Pheno (Accelerate Diagnostics, Tucson, AZ), which tests for eight different targets (Escherichia coli, Klebsiella spp., Enterobacter spp., Proteus spp., Citrobacter spp., Serratia marcescens, Pseudomonas aeruginosa, and Acinetobacter baumannii) and additionally provides AST for some of the most commonly administered antibiotics, is used. Last, for yeasts in the blood, the PNA-FISH test (OpGen AdvanDx, Gaithersburg, MD) is performed with dual (Candida albicans/Candida glabrata) and traffic light (C. albicans/Candida parapsilosis, C. glabrata/Candida krusei, Candida tropicalis) probes. The respective turnaround times (time on the instrument) are 1 h for the Xpert MRSA/SA assay, 7 h for the Accelerate Pheno (2 h for ID and 7 h for both ID and AST), and 90 min for the PNA-FISH. However, due to the need for a technologist’s microscopic review, the PNA-FISH procedure involves increased hands-on time, leading to a total of 4 h on average, counting from the beginning of the processing to result. Moreover, PNA-FISH is primarily done on the first shift Monday through Friday and if a blood culture flags positive with yeasts outside this window, MALDI-TOF MS is set up to identify the yeast from the colony.

Apart from the mRDT, for every positive blood culture, the blood is inoculated onto a specific solid agar medium according to the Gram stain and, once there is colony growth, identification is performed using MALDI-TOF MS (Vitek MS; bioMérieux, Durham NC) or the Vitek 2 automated system (bioMérieux) as appropriate. The Vitek 2 is used for identification only in cases where a result from MALDI-TOF MS is inconclusive. Regarding AST, SOC testing includes the Vitek 2 system and Sensititre (Thermo Scientific, Waltham MA) for nonfermentative Gram-negative rods and yeasts. The algorithm implemented in our laboratory for positive blood cultures is depicted in Fig. 1.

### The GenMark Dx ePlex blood culture identification panel testing.

All three BCID panels were used in our study: the BCID Gram-positive panel (BCID-GP) for Gram-positive bacteria, the BCID Gram-negative panel (BCID-GN) for Gram-negative bacteria, and the BCID fungal pathogen panel (BCID-FP) for yeasts/fungi (14, 23, 24). For every blood culture, a panel was selected based on the Gram stain result. The positive blood cultures were run on the ePlex usually within 24 h of culture positivity. The study experiments were not conducted on the weekends, and therefore, some positive bottles were run on the ePlex within 24 to 72 h, which was permissible according to the manufacturer’s package insert. The BCID panels were handled and used according to the manufacturer’s package insert. After withdrawing the specimen from each blood culture bottle using standard aseptic technique, 50 μL of positive blood culture was loaded with the use of a pipette into the cartridge, and the cartridge was loaded into an available bay of the ePlex instrument. Upon a positive pan target, which is available on all three BCID panels and indicates that an organism(s) not picked by the in-use panel is present, the implied panel was also run in order to identify all organisms found in a blood culture. In the instance of an invalid result, the sample was repeated once to rule out potential cartridge issues.

### Data analysis.

The performance of the ePlex BCID panels was not compared to a single reference method (since more than one method was used to identify most of the organisms), and thus, positive percent agreement (PPA) and negative percent agreement (NPA) were calculated based on the result(s) from SOC testing. All specimens with a discrepant result between ePlex BCID panels and SOC testing were further analyzed with a third method, which included one of the following: *mecA* PCR for methicillin-resistant S. aureus, chromogenic agar for C. albicans, and sequencing for the remainder of the discrepant organisms. Discordancy resolution was performed at GenMark Dx for research purposes only by performing further BCID panel testing, growth on selective medium, and PCR/sequencing.

Subsequently, the internal laboratory information system was accessed and the following data were extracted and entered in an Excel spreadsheet for all included positive blood cultures: time of bottle positivity, time the Gram stain result was called to the provider, and time to result for any method, which was counted from the reading of the Gram stain to the calling of the final identification of the organism to the provider. Then, the median time to result and interquartile range values were calculated for all identification methods (Vitek-MS/MALDI-TOF MS, Xpert MRSA/SA, Accelerate Pheno, and PNA-FISH). The turnaround time for the ePlex BCID panels was ~90 min, but a statistical comparison of time to result between the ePlex and other assays could not be performed because we did not have real-life time-to-result data for the ePlex.
